# Increased risk of venous thromboembolism in children and teenagers with inflammatory bowel disease: a systematic review and meta-analysis

**DOI:** 10.7717/peerj.21056

**Published:** 2026-04-01

**Authors:** Yuezhong Shen, Yuyue Jiang, Yan Wang, Changqing Xu, Xi Wang, Xuqing Huang

**Affiliations:** Department of Respiratory and Critical Care Medicine, Affiliated Hospital of Hangzhou Normal University, Hangzhou, Zhejiang Province, China

**Keywords:** Inflammatory bowel disease, Children, Teenagers, Venous thromboembolism, Systematic review

## Abstract

**Background:**

The increased risk of venous thromboembolism (VTE) in adults with inflammatory bowel disease (IBD) is well known, but we know less about the VTE risk of IBD in children and teenagers. We evaluated the risk of VTE in children and teenagers through systematic review and meta-analysis.

**Methods:**

A systematic search was conducted in PubMed, Embase, Cochrane Library, Web of Science for studies from the establishment of these databases to February 10, 2026, to find relevant research on the risk of VTE in children and teenagers with IBD (PROSPERO, ID: CRD420251081653). Random-effects and fixed-effects models were used to estimate the relative risk (RR) and the corresponding 95% confidence interval (CI). The quality of the included studies was evaluated using the Newcastle-Ottawa Scale.

**Results:**

Seven cohort studies were included in this systematic review, involving 101,253 children and teenagers with IBD and 19,651,587 non-IBD controls. Compared with non-IBD children and teenagers, the overall RR of VTE in children and teenagers with IBD was 6.94 (95% CI [1.87–25.69], *P* = 0.004). The risk of deep vein thrombosis (DVT) in children and teenagers with IBD was increased (RR = 6.25, 95% CI [1.13–34.69], *P* = 0.036); the risk of pulmonary thromboembolism (PE) in children and teenagers with IBD was also increased, but there was no statistically significant difference (RR = 3.13, 95% CI [0.96–10.19], *P* = 0.058). Children and teenagers with ulcerative colitis (UC) have a higher risk of VTE (RR = 7.53, 95% CI [2.97–19.10], *P* < 0.001) than those with Crohn’s disease (CD) (RR = 3.69, 95% CI [1.67–8.17], *P* = 0.001). The unadjusted VTE risk in children and teenagers with IBD (RR = 8.19, 95% CI [2.29–29.32], *P* = 0.001) was higher than the adjusted VTE risk for confounding factors (RR = 4.53, 95% CI [1.91–10.77], *P* = 0.001).

**Conclusions:**

Children and teenagers with IBD are at a significantly increased risk of developing VTE. Therefore, VTE prevention strategies should be emphasized in this population as well as in adults.

## Introduction

Venous thromboembolism (VTE) encompasses two major clinical entities: deep vein thrombosis (DVT) and pulmonary embolism (PE). Although the mortality rate associated with VTE has declined in recent years, the reduction has been relatively slow (Giorgio et al., 2023; Zuin et al., 2024). Public awareness of VTE remains limited (Lin et al., 2023; Potere et al., 2024; Wendelboe & Weitz, 2024). Moreover, the recurrence of VTE, its chronic complications, and bleeding events related to anticoagulant therapy impose considerable physical and psychological burdens on patients (Oleksiuk-Bójko & Lisowska, 2023). VTE occurs more frequently in the elderly population (Tritschler & Aujesky, 2017). However, with improvements in socioeconomic conditions, increasing attention has been directed toward the prevention and management of VTE in children and teenagers. Most children experiencing VTE present risk factors, such as central venous catheterization, cancer, severe infection, congenital cardiopathy, serious trauma, premature birth (Monagle et al., 2025). The distribution of risk factors for VTE patients varies with different ages of onset. In younger patients, central venous catheterization, hereditary thrombophilia, a positive family history of VTE, and oral contraceptive use are more common (Citla Sridhar, Abou-Ismail & Ahuja, 2020; Linnemann et al., 2014). In adults, these conditions are more likely linked to cancer (Linnemann et al., 2014). In addition, the relative risk (RR) of VTE in children with congenital cardiopathy appears to be higher than that in adults (Cuszynska-Kruk et al., 2024). Premature birth increases the risk of VTE in children (Zöller et al., 2014), but its effect in adulthood is unclear.

Inflammatory bowel disease (IBD) is a gastrointestinal disorder characterized by chronic inflammation of the digestive tract, encompassing ulcerative colitis (UC) and Crohn’s disease (CD) (Torres et al., 2017; Ungaro et al., 2017). IBD can affect individuals of any age (Sarter et al., 2024). Although its exact etiology remains unclear, IBD is thought to be influenced by a combination of genetic predisposition, environmental factors, and immune system dysfunction (Guan, 2019; Rosen, Dhawan & Saeed, 2015; Zhang & Li, 2014). Patients with IBD frequently present with various extraintestinal manifestations, including VTE (Fumery et al., 2014; Peyrin-Biroulet et al., 2011). This is likely due to the common hypercoagulable state observed in IBD (Lagrange et al., 2021; Middeldorp, 2023). The RR of VTE in adults with IBD is approximately double that of the general population (Arvanitakis et al., 2021; Yuhara et al., 2013). In recent decades, the incidence and prevalence of IBD in younger populations have risen sharply worldwide (Chen et al., 2024; Khan, Kuenzig & Benchimol, 2023; Kuenzig et al., 2022; Long et al., 2024). However, the risk of VTE in children and teenager IBD patients remains less well understood.

We conducted a systematic review and meta-analysis to synthesize recent evidence and clarify the relative risk of VTE in children and adolescents with IBD. Our objective is to provide stronger evidence to inform the clinical prevention and management of VTE in this age group.

## Materials and Methods

This study conducts a systematic review based on the Preferred Reporting Items for Systematic Reviews and Meta-Analyses (PRISMA) (Method S1) (Page et al., 2021). The protocol has been registered in the international prospective register of systematic reviews, PROSPERO (Protocol ID: CRD420251081653).

### Study eligibility

Inclusion criteria: (1) The study population includes children and teenagers diagnosed with IBD (CD, UC, or undifferentiated IBD); (2) non-IBD children and teenagers are used as controls; (3) the study design is a cohort study; (4) the study outcomes are related to VTE in children or teenagers with IBD; (5) the language of the study is English; (6) the ages of the subjects in both groups were 20 years old or younger. Exclusion criteria: (1) The study subjects were either non-IBD patients or only adult IBD patients; (2) the study design is a case-control study or a cross-sectional study. (3) the study outcomes are not related to VTE; (4) studies without data or data that cannot be extracted.

### Search strategy

A search was conducted in PubMed, Embase, Cochrane Library and Web of Science databases from the establishment of these databases until February 10, 2026. We also searched the references of the included studies and the published meta-analyses to identify additional relevant literature. Search using Medical Subject Headings (MeSH) and text words: inflammatory bowel disease, ulcerative colitis, Crohn’s disease, venous thromboembolism, children (detailed search strategy can be found in Method S2).

### Study selection and data capture

Two researchers (SYZ and WY) independently conducted the literature screening and data extraction from February 10 to February 12, 2026. In case of any disagreement, a third party was consulted for a decision (JYY). The extracted data included: authors and publication year, study design, period, source of the population, number of participants, incidence of VTE, IBD type, and controlled confounding factors. The extracted effect sizes included RR, odds ratio (OR) or hazard ratio (HR), along with their 95% confidence intervals (CI). If the above data is not reported, the original data will be used for calculation. If confounding factors were adjusted for, the effect sizes adjusted for these factors were prioritized. If a study reports results for multiple age groups, we only extract the data of the children and teenagers subgroup that best meet the inclusion criteria.

### Study quality

Two researchers (SYZ and WY) assessed the quality of the studies in the research using the Newcastle-Ottawa Scale (NOS). In case of any disagreement, a third party was consulted for a decision (JYY). This scale evaluates studies based on three categories: selection, comparability and outcome. The higher the score, the higher the quality of the research.

### Statistical analyses

Meta-analysis was performed using Stata statistical software version 14.0 (Stata Corp, College Station, TX, USA). Heterogeneity was analyzed using the I^2^ test. If *P* ≥ 0.10 and I^2^ ≤ 50%, no significant heterogeneity was considered, and a fixed-effects model was used for pooling; otherwise, a random-effects model was applied. RR was used as the effect size, with a 95% CI reported. A *P*-value < 0.05 was considered statistically significant. Sensitivity analysis was conducted using a leave-one-out method. Potential publication bias was assessed through visual evaluation of a funnel plot and verified using Egger’s test.

## Results

### Eligible studies

**Figure 1:**
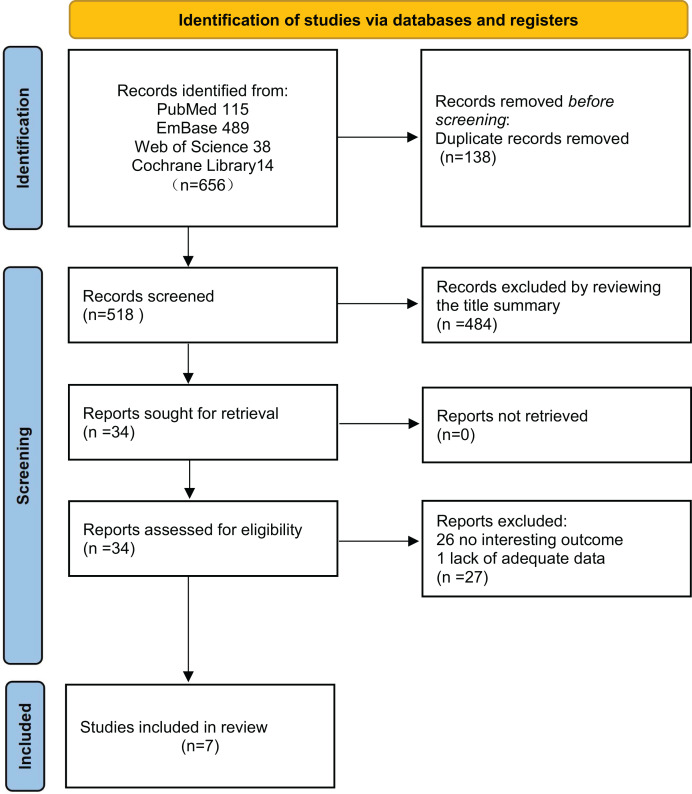
Study selection flow diagram presented according to the PRISMA statement.

A total of 656 articles were identified (Fig. 1). After removing duplicates, 518 articles were included. After reading the titles and abstracts, 34 articles were selected for full-text review. Among these, 26 articles were excluded due to irrelevant outcomes, and one article was excluded because the data could not be extracted. Ultimately, seven articles (Cairo et al., 2018; Harvey et al., 2025; Kappelman et al., 2011; Kim et al., 2022; Kuenzig et al., 2021; Nguyen & Sam, 2008; Nylund et al., 2013) were assessed for quality and all were included in the final analysis.

### General characteristics

Table 1 summarizes the general characteristics of the included studies. All seven studies included in this research were population-based cohort studies. The data were sourced from national domestic registries of various countries. The ages of the subjects in all included studies were 20 years old or younger. Only the 0~19 years subgroup estimate from Kim et al. (2022) was extracted and included in the meta-analysis (and that controls correspond to the same age band). A total of 101,253 children and teenagers with IBD and 19,651,587 non-IBD controls were included. Three studies (Kappelman et al., 2011; Kuenzig et al., 2021; Nylund et al., 2013) compared the risk of DVT or PE between children and teenagers with IBD and the controls. Four studies (Kim et al., 2022; Kuenzig et al., 2021; Nguyen & Sam, 2008; Nylund et al., 2013) compared the risk of VTE in children and teenagers with different types of IBD. Three studies (Kappelman et al., 2011; Kim et al., 2022; Kuenzig et al., 2021) adjusted for confounding factors, including age, gender, Malignancy, and surgery, *etc*.

**Table 1:** Study characteristics.

Study, Year [Ref.]	Country	Study design	Period	Age (years)	Data source	IBD (*n*)	Incidence of VTE (%)	Controls (*n*)	Incidence of VTE(%)	Patient	Confounders for adjustment
Nguyen & Sam (2008)	USA	Cohort	1998–2004	0~20	Nationwide Inpatient Sample database	12,432	0.49	103,989	0.07	UC, CD	NA
Kappelman et al. (2011)	Denmark	Cohort	1980–2007	0~20	Danish National Patient Registry	5,424	0.74	49,113	0.17	UC, CD, IBD-U	Malignancy, Surgery, Fracture and Pregnancy
Nylund et al. (2013)	USA	Cohort	1997–2009	5~20	Healthcare Cost and Utilization Project Kids’ Inpatient Database	68,394	1.18	7,379,898	0.50	UC, CD	NA
Cairo et al. (2018)	USA	Cohort	2012–2015	0~19	National Surgical Quality Improvement Program-Pediatric database	410	0.98	34,403	0.19	IBD	NA
Kuenzig et al. (2021)	Canada	Cohort	2001–2018	0~16	Health administrative data from five Canadian provinces	3,593	5.61	16,284	0.14	UC, CD, IBD-U	Age, Sex, Income and Rurality
Kim et al. (2022)	South Korea	Cohort	2006–2015	0~19	National Health Insurance data	6,251	0.34	18,743	0.13	UC, CD	Age, sex, socioeconomic status, residence, and comorbidities
Harvey et al. (2025)	England	Cohort	2001–2019	0~18	Hospital Episode Statistics	4,749	1.79	12,049,157	0.03	UC, CD	NA

**Note:**

USA, The United States; IBD, inflammatory bowel disease; UC, ulcerative colitis; CD, Crohn’s disease; IBD-U, inflammatory bowel disease-unclassified; VTE, Venous thromboembolism; N/A, not available.

### Quality assessment

We assessed the quality of the seven studies using the NOS. Three studies were rated eight stars and four studies were rated six stars (Table S1).

### The main results of the meta-analysis

**Figure 2:**
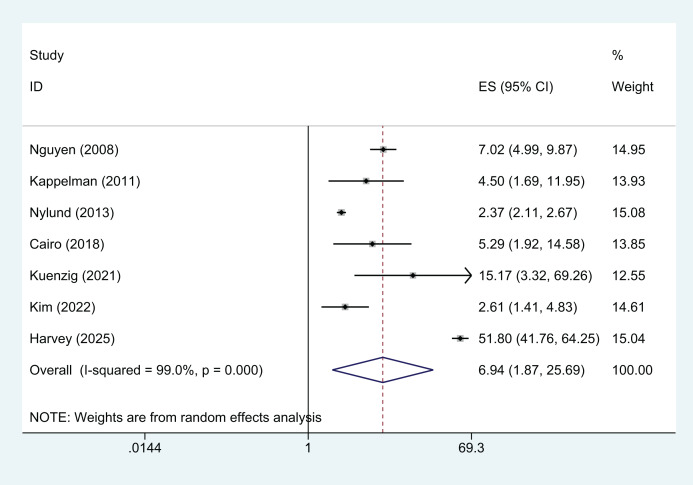
Forest plot demonstrating the summary relative risk for venous thromboembolic events in children and teenagers with inflammatory bowel disease. The size of squares is proportional to the weight of each study with the error bar showing the 95% CI. Diamond represents the pooled estimate of RR with the 95% CI. RR, relative risk; CI, confidence interval; ES, effect size, here it refers to RR.

Compared with children and teenagers without IBD, the overall RR of VTE in children and teenagers with IBD is 6.94 (95% CI [1.87–25.69], *P* = 0.004), with significant heterogeneity (I^2^ = 99%) (Fig. 2). Sensitivity analysis confirmed that the combined effect size was not influenced by any single study, indicating that the results of our study were relatively stable (Sensitivity analysis can be found in Fig. S1).

### The results of subgroup analysis

To clarify the source of heterogeneity, we also performed subgroup analyses based on the type of outcome (Table 2). The results showed that the risk of DVT in children and teenagers with IBD was increased (RR = 6.25, 95% CI [1.13–34.69], *P* = 0.036), with heterogeneity (I^2^ = 93%); the risk of PE in children and teenagers with IBD was also increased, but there was no statistically significant difference (RR = 3.13, 95% CI [0.96–10.19], *P* = 0.058). Additionally, the risk of VTE in UC was increased (RR = 7.53, 95% CI [2.97–19.10], *P* < 0.001), with heterogeneity (I^2^ = 75%); the risk of VTE in CD was increased (RR = 3.69, 95% CI [1.67–8.17], *P* = 0.001), with heterogeneity (I^2^ = 88%). After adjusting for confounding factors, the risk of VTE in children and teenagers with IBD was increased (RR = 4.53, 95% CI [1.91–10.77], *P* = 0.001), with heterogeneity (I^2^ = 57%); studies that did not adjust for confounding factors indicated, children and teenagers with IBD have a higher risk of VTE (RR = 8.19, 95% CI [2.29–29.32], *P* = 0.001), with heterogeneity (I^2^ = 98%).

**Table 2:** Results of meta-analyses by type of outcome.

Type of outcome	Included studies	RR (95% CI)	Heterogeneity		Significance test	
			I^2^	*P*	Z	*P*-values
DVT in IBD	3	6.25 [1.13–34.69]	93%	<0.001	2.10	0.036
PE in IBD	3	3.13 [0.96–10.19]	58%	0.094	1.90	0.058
VTE in UC	4	7.53 [2.97–19.10]	75%	0.007	4.25	<0.001
VTE in CD	4	3.69 [1.67–8.17]	88%	<0.001	3.22	0.001
Adjusted VTE risk	3	4.53 [1.91–10.77]	57%	0.096	3.42	0.001
Unadjusted VTE risk	7	8.19 [2.29–29.32]	98%	<0.001	3.23	0.001

**Note:**

IBD, inflammatory bowel disease; DVT, deep vein thrombosis; PE, pulmonary embolism; VTE, venous thromboembolism; UC, ulcerative colitis; CD, Crohn’s disease; RR, relative risk; CI, confidence interval.

### Publication bias

**Figure 3:**
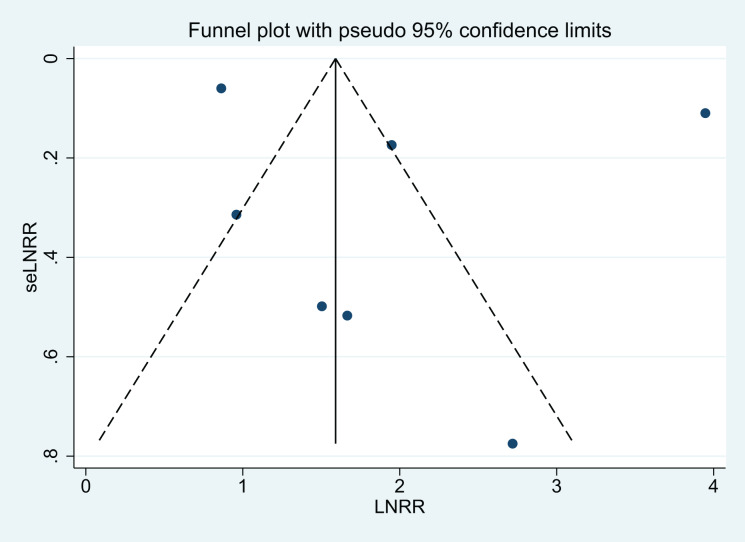
Funnel chart. RR, relative risk.

The visual result of the funnel chart (Fig. 3) and the results of Egger’s regression test (*P* = 0.597) shows that no clear evidence of publication bias was detected; however, assessment is limited by the small number of studies.

## Discussion

### Main findings

This meta-analysis included seven cohort studies involving 101,253 children and teenagers diagnosed with IBD. The analysis revealed that the risk of VTE in these children and teenagers is 6.94 times higher compared to those without IBD. The risk varies depending on the specific type of IBD and the nature of the VTE.

### Interpretation of findings

A previous meta-analysis reported a pooled incidence rate of VTE of 0.02 among children and teenagers with IBD, with a RR of 2.99 (Zhang et al., 2022). However, that analysis included only two studies and did not perform any subgroup analyses. In contrast, our meta-analysis incorporated both previously omitted and newly published studies, thereby updating the estimated relative risk of VTE in children and teenagers. Furthermore, we conducted subgroup analyses according to the type of IBD and category of VTE. Our findings suggest that the relative risk of VTE among children and teenagers with IBD may have been previously underestimated.

The exact mechanism of VTE in patients with IBD remains unclear, but it may be associated with inflammation, thrombocytosis, and alterations in the coagulation system. Inflammation can trigger the coagulation cascade, such as the increase in interleukin-6, which promotes coagulation while inhibiting fibrinolysis (de Maat et al., 2014; Xi et al., 2023). Patients with IBD may develop reactive thrombocytosis through the CD40 and protease-activated receptor (PAR) pathways (Schmid et al., 2014; Yazici et al., 2010). Both thrombocytosis and increased platelet reactivity may contribute to thrombosis. Additionally, the increase in thrombin-antithrombin complex (TAT), coagulation factor V (FV), and fibrinogen associated with IBD may be linked to VTE (Lagrange et al., 2021).

Our research indicates that the relative risk of VTE in children and teenagers with IBD appears to be higher than that in adults (Kappelman et al., 2011; Nguyen & Sam, 2008). This remains the case even after adjusting for risk factors such as malignancy and surgery. This elevated risk may be attributed to the generally low incidence of VTE in younger populations (Nguyen & Sam, 2008). Given that VTE risk is typically lower in younger individuals within the general population, it may be underappreciated in IBD patients. As a result, it is crucial to assess the VTE risk in children and teenagers with IBD with the same level of vigilance applied to adults. Several factors in IBD patients may influence VTE incidence. In adults, corticosteroids, pregnancy, blood transfusions, hypoproteinemia, and total parenteral nutrition are recognized risk factors for VTE (Gozdzik et al., 2022, 2025; Kim et al., 2019; Zhang & Wang, 2021). In children and teenagers with IBD, VTE may be related to the following factors: female, active disease, immunosuppressant, biologics, systemic steroids, oral contraceptive pills and central venous catheter (Bence et al., 2020; De Laffolie et al., 2022; Kumar et al., 2017; Richard et al., 2025; Zitomersky et al., 2013).

In the subgroup analysis, we observed that children and teenagers with IBD exhibit a higher risk of DVT compared to PE, a pattern also seen in adults (Arvanitakis et al., 2021). This may be attributed to the fact that PE often develops as a complication of DVT. Several studies have indicated that untreated patients with proximal DVT have a 50% chance of developing symptomatic PE within 3 months (Di Minno et al., 2016; Moheimani & Jackson, 2011). The latest meta-analysis (Gozdzik et al., 2025) indicates that the risk of VTE in adults with UC and CD is higher than in the general population, with a more pronounced increase observed in UC patients. Previous meta-analyses on children and teenagers (Zhang et al., 2022) did not differentiate VTE risk by IBD type, but instead focused on summarizing VTE incidence. The incidence was 0.05 in UC patients, which is higher than the 0.02 in CD patients. However, direct comparability between the UC and CD subgroups in these studies is lacking. Our meta-analysis confirms that children and teenagers with UC or CD have a higher VTE risk compared to non-IBD patients. UC patients, in particular, exhibit a significantly higher RR, similar to that observed in adults. The reasons for the higher VTE risk in UC patients compared to CD patients remain unclear. Kuenzig et al. (2021) suggest that UC patients may be more prone to thrombosis due to severe inflammation. Additionally, bleeding complications reduce the effectiveness of VTE prevention in UC patients. Other studies have shown that severe UC often leads to anemia and reactive thrombocytosis, which could contribute to thrombosis (Papa et al., 2014; Yan et al., 2013).

Besides IBD, other diseases like systemic lupus erythematosus and rheumatoid arthritis have also been linked to VTE (Bello et al., 2023; Fazal et al., 2024). Further investigation into this link in young individuals is needed.

### Limitations

Our study has several limitations. Age is an independent risk factor for VTE, including in patients with IBD. Two cohort studies (Kappelman et al., 2011; Nguyen & Sam, 2008) have shown that although children and teenagers with IBD have a higher RR of VTE, their actual absolute risk of VTE is not higher than that of adults. VTE screening and prevention in IBD patients should involve individualized assessments, taking both relative and absolute risks into account. This meta-analysis demonstrated substantial heterogeneity, likely due to differences in population characteristics across studies. Factors such as active disease and the use of systemic corticosteroids are known to be associated with increased VTE risk in IBD patients; patients recently diagnosed with IBD may experience a peak in inflammatory burden due to the treatment just beginning, thereby increasing the risk of VTE (Harvey et al., 2025). However, insufficient data from available studies precluded further analysis of these factors. VTE diagnosis is challenging, and misclassification may have contributed to the observed heterogeneity. Although no publication bias was detected, the relatively small number of studies included in the analysis could have reduced statistical power, potentially leading to false-positive or false-negative results.

## Conclusions

Children and teenagers with IBD are at a significantly increased risk of developing VTE. Therefore, VTE prevention strategies should be emphasized in this population as well as in adults. Further high-quality, large-scale studies are warranted to better define the VTE risk in younger IBD patients, particularly to compare their absolute risk with that of adults.

## Supplemental Information

10.7717/peerj.21056/supp-1 Supplemental Information 1 Raw Data.

10.7717/peerj.21056/supp-2 Supplemental Information 2 The Newcastle-Ottawa Scale used to assess the quality of the included studies.

10.7717/peerj.21056/supp-3 Supplemental Information 3 Results of sensitivity analysis.

10.7717/peerj.21056/supp-4 Supplemental Information 4 PRISMA 2020 checklist.

10.7717/peerj.21056/supp-5 Supplemental Information 5 Search strategy.

10.7717/peerj.21056/supp-6 Supplemental Information 6 Rationale and Contribution.

## Additional Information and Declarations

### Data Availability

The following information was supplied regarding data availability:

This is a systematic review/meta-analysis.
