# Supplementary material for: Increased risk of venous thromboembolism in children and teenagers with inflammatory bowel disease: a systematic review and meta-analysis
Source: PeerJ. 2026 Apr 1;14:e21056. doi: 10.7717/peerj.21056 (PMC13050219; doi:10.7717/peerj.21056)
Supplement: Supplemental Information 2 [file peerj-14-21056-s002.docx]

**Supplemental Table S1 The Newcastle-Ottawa Scale used to assess the quality of the included studies**

| Study,Year  [Ref.] | Selection | | | | Comparability | | Outcome | | | Overall quality score |
| --- | --- | --- | --- | --- | --- | --- | --- | --- | --- | --- |
| Quality  assessment  criteria | Representativeness of  exposed  cohort | Selection of  non−  exposed  cohort | Ascertainment  of exposure | Demonstration  that outcome  of interest was  not present at  start of study | Adjust for  the most  important  risk factors | Adjust  for  other  risk  factors | Assessment  of outcome | Follow−up  length | Loss to  follow−up  rate |  |
| Kuenzig 2021 | ★ | ★ | ★ | - | - | - | ★ | ★ | ★ | 6 |
| Cairo  2018 | ★ | ★ | ★ | - | ★ | ★ | ★ | ★ | ★ | 8 |
| Nylund 2013 | ★ | ★ | ★ | - | - | - | ★ | ★ | ★ | 6 |
| Kappelman 2011 | ★ | ★ | ★ | - | - | - | ★ | ★ | ★ | 6 |
| Nguyen 2008 | ★ | ★ | ★ | - | ★ | ★ | ★ | ★ | ★ | 8 |
| Kim  2022 | ★ | ★ | ★ | - | ★ | ★ | ★ | ★ | ★ | 8 |
| Harvey 2025 | ★ | ★ | ★ | - | - | - | ★ | ★ | ★ | 6 |
