# Supplementary material for: Increased risk of venous thromboembolism in children and teenagers with inflammatory bowel disease: a systematic review and meta-analysis
Source: PeerJ. 2026 Apr 1;14:e21056. doi: 10.7717/peerj.21056 (PMC13050219; doi:10.7717/peerj.21056)
Supplement: Supplemental Information 5 [file peerj-14-21056-s005.docx]

**Supplemental Method 2.** **Search strategy**

**PUBMED:**

| Search Number | Search Description |
| --- | --- |
| #1 | "Inflammatory Bowel Diseases"[Mesh] OR Inflammatory Bowel Disease[Title/Abstract] OR Inflammatory Bowel Diseases[Title/Abstract] OR Bowel Diseases, Inflammatory[Title/Abstract] OR Bowel Disease, Inflammatory[Title/Abstract] OR "Crohn Disease"[Mesh] OR Crohn Disease[Title/Abstract] OR "Colitis, Ulcerative"[Mesh] OR Colitis, Ulcerative[Title/Abstract] |
| #2 | "Child"[Mesh] OR "Pediatrics"[Mesh] OR "Adolescent"[Mesh] OR Child[Title/Abstract] OR Children[Title/Abstract] OR Pediatrics[Title/Abstract] OR Pediatric[Title/Abstract] OR Adolescent[Title/Abstract] OR Adolescents[Title/Abstract] OR Adolescence[Title/Abstract] OR Adolescents, Female[Title/Abstract] OR Adolescent, Female[Title/Abstract] OR Female Adolescent[Title/Abstract] OR Female Adolescents[Title/Abstract] OR Adolescents, Male[Title/Abstract] OR Adolescent, Male[Title/Abstract] OR Male Adolescent[Title/Abstract] OR Male Adolescents[Title/Abstract] OR Youth[Title/Abstract] OR Youths[Title/Abstract] OR Teens[Title/Abstract] OR Teen[Title/Abstract] OR Teenagers[Title/Abstract] OR Teenager[Title/Abstract] |
| #3 | ("Pulmonary Embolism"[Mesh]) OR (Pulmonary Embolism[Title/Abstract] OR Embolism, Pulmonary[Title/Abstract] OR Embolisms, Pulmonary[Title/Abstract] OR Pulmonary Embolisms[Title/Abstract] OR Pulmonary Thromboembolisms[Title/Abstract] OR Thromboembolisms, Pulmonary[Title/Abstract] OR Pulmonary Thromboembolism[Title/Abstract] OR Thromboembolism, Pulmonary[Title/Abstract]) OR ((((("Venous Thromboembolism"[Mesh]) OR (Venous Thromboembolism[Title/Abstract] OR Thromboembolism, Venous[Title/Abstract] OR VTE[Title/Abstract])) OR ("Thromboembolism"[Mesh])) OR (Thromboembolism[Title/Abstract] OR Thromboembolisms[Title/Abstract])) OR ("Venous Thrombosis"[Mesh])) OR (Venous Thrombosis[Title/Abstract] OR Phlebothrombosis[Title/Abstract] OR Phlebothromboses[Title/Abstract] OR Thrombosis, Venous[Title/Abstract] OR Thromboses, Venous[Title/Abstract] OR Venous Thromboses[Title/Abstract] OR Deep Vein Thrombosis[Title/Abstract] OR Deep Vein Thromboses[Title/Abstract] OR Thromboses, Deep Vein[Title/Abstract] OR Vein Thromboses, Deep[Title/Abstract] OR Vein Thrombosis, Deep[Title/Abstract] OR Deep Venous Thrombosis[Title/Abstract] OR Deep Venous Thromboses[Title/Abstract] OR Thromboses, Deep Venous[Title/Abstract] OR Thrombosis, Deep Venous[Title/Abstract] OR Venous Thromboses, Deep[Title/Abstract] OR Venous Thrombosis, Deep[Title/Abstract] OR Deep-Vein Thrombosis[Title/Abstract] OR Deep-Vein Thromboses[Title/Abstract] OR Thromboses, Deep-Vein[Title/Abstract] OR Thrombosis, Deep-Vein[Title/Abstract] OR Deep-Venous Thrombosis[Title/Abstract] OR Deep-Venous Thromboses[Title/Abstract] OR Thromboses, Deep-Venous[Title/Abstract] OR Thrombosis, Deep-Venous[Title/Abstract] OR Thrombosis, Deep Vein[Title/Abstract] OR "Thrombosis"[Mesh] OR Thrombosis[Title/Abstract] OR Thromboses[Title/Abstract] OR Thrombus[Title/Abstract] OR Blood Clot[Title/Abstract] OR Blood Clots[Title/Abstract] OR Atherothrombosis[Title/Abstract]) |
| #4 | (((((("Case-Control Studies"[Mesh]) OR ("Cohort Studies"[Mesh])) OR ("Retrospective Studies"[Mesh])) OR ("Longitudinal Studies"[Mesh])) OR ("Follow-Up Studies"[Mesh])) OR (Case-Control Studies[Title/Abstract] OR Case-Control Study[Title/Abstract] OR Studies, Case-Control[Title/Abstract] OR Study, Case-Control[Title/Abstract] OR Case-Comparison Studies[Title/Abstract] OR Case Comparison Studies[Title/Abstract] OR Case-Comparison Study[Title/Abstract] OR Studies, Case-Comparison[Title/Abstract] OR Study, Case-Comparison[Title/Abstract] OR Case Control Studies[Title/Abstract] OR Case Control Study[Title/Abstract] OR Studies, Case Control[Title/Abstract] OR Study, Case Control[Title/Abstract] OR Matched Case-Control Studies[Title/Abstract] OR Case-Control Studies, Matched[Title/Abstract] OR Case-Control Study, Matched[Title/Abstract] OR Matched Case Control Studies[Title/Abstract] OR Matched Case-Control Study[Title/Abstract] OR Studies, Matched Case-Control[Title/Abstract] OR Study, Matched Case-Control[Title/Abstract] OR Cohort Studies[Title/Abstract] OR Cohort Study[Title/Abstract] OR Studies, Cohort[Title/Abstract] OR Study, Cohort[Title/Abstract] OR Retrospective Studies[Title/Abstract] OR Studies, Retrospective[Title/Abstract] OR Study, Retrospective[Title/Abstract] OR Retrospective Study[Title/Abstract] OR Longitudinal Studies[Title/Abstract] OR Longitudinal Study[Title/Abstract] OR Studies, Longitudinal[Title/Abstract] OR Study, Longitudinal[Title/Abstract] OR Longitudinal Survey[Title/Abstract] OR Longitudinal Surveys[Title/Abstract] OR Survey, Longitudinal[Title/Abstract] OR Surveys, Longitudinal[Title/Abstract] OR Follow-Up Studies[Title/Abstract] OR Follow Up Studies[Title/Abstract] OR Follow-Up Study[Title/Abstract] OR Studies, Follow-Up[Title/Abstract] OR Study, Follow-Up[Title/Abstract] OR Followup Studies[Title/Abstract] OR Followup Study[Title/Abstract] OR Studies, Followup[Title/Abstract] OR Study, Followup[Title/Abstract])) |
| #5 | #1 AND #2 AND #3 AND #4 |

**EmBase:**

| Search Number | Search Description |
| --- | --- |
| #1 | 'inflammatory bowel disease'/exp OR 'inflammatory bowel disease':ab,ti OR 'bowel diseases, inflammatory':ab,ti OR 'inflammatory bowel diseases':ab,ti OR 'bowel disease, inflammatory':ab,ti OR 'crohn disease'/exp OR 'crohn disease':ab,ti OR 'ulcerative colitis'/exp OR 'ulcerative colitis':ab,ti |
| #2 | 'Child'/exp OR 'Pediatrics'/exp OR 'Child':ab,ti OR 'Children':ab,ti OR 'Pediatrics':ab,ti OR 'Pediatric':ab,ti OR 'Adolescent':ab,ti OR 'Adolescents':ab,ti OR 'Adolescence':ab,ti OR 'Adolescents, Female':ab,ti OR 'Adolescent, Female':ab,ti OR 'Female Adolescent':ab,ti OR 'Female Adolescents':ab,ti OR 'Adolescents, Male':ab,ti OR 'Adolescent, Male':ab,ti OR 'Male Adolescent':ab,ti OR 'Male Adolescents':ab,ti OR 'Youth':ab,ti OR 'Youths':ab,ti OR 'Teens':ab,ti OR 'Teen':ab,ti OR 'Teenagers':ab,ti OR 'Teenager':ab,ti |
| #3 | 'lung embolism'/exp OR 'Pulmonary Embolism':ab,ti OR 'Embolism, Pulmonary':ab,ti OR 'Embolisms, Pulmonary':ab,ti OR 'Pulmonary Embolisms':ab,ti OR 'Pulmonary Thromboembolisms':ab,ti OR 'Thromboembolisms, Pulmonary':ab,ti OR 'Pulmonary Thromboembolism':ab,ti OR 'Thromboembolism, Pulmonary':ab,ti OR 'venous thromboembolism'/exp OR 'venous thromboembolism':ab,ti OR 'thromboembolism, venous':ab,ti OR 'vte':ab,ti OR 'thromboembolism'/exp OR 'thromboembolism':ab,ti OR 'thromboembolisms':ab,ti OR 'vein thrombosis'/exp OR 'venous thrombosis':ab,ti OR 'phlebothrombosis':ab,ti OR 'phlebothromboses':ab,ti OR 'thrombosis, venous':ab,ti OR 'thromboses, venous':ab,ti OR 'venous thromboses':ab,ti OR 'deep vein thrombosis':ab,ti OR 'deep vein thromboses':ab,ti OR 'thromboses, deep vein':ab,ti OR 'vein thromboses, deep':ab,ti OR 'vein thrombosis, deep':ab,ti OR 'deep venous thrombosis':ab,ti OR 'deep venous thromboses':ab,ti OR 'thromboses, deep venous':ab,ti OR 'thrombosis, deep venous':ab,ti OR 'venous thromboses, deep':ab,ti OR 'venous thrombosis, deep':ab,ti OR 'deep-vein thrombosis':ab,ti OR 'deep-vein thromboses':ab,ti OR 'thromboses, deep-vein':ab,ti OR 'thrombosis, deep-vein':ab,ti OR 'deep-venous thrombosis':ab,ti OR 'deep-venous thromboses':ab,ti OR 'thromboses, deep-venous':ab,ti OR 'thrombosis, deep-venous':ab,ti OR 'thrombosis, deep vein':ab,ti OR 'dvt':ab,ti OR 'thrombosis'/exp OR 'thrombosis':ab,ti OR 'thromboses':ab,ti OR 'thrombus':ab,ti OR 'blood clot':ab,ti OR 'blood clots':ab,ti OR 'atherothrombosis':ab,ti |
| #4 | 'case control study'/exp OR 'cohort analysis'/exp OR 'retrospective study'/exp OR 'longitudinal study'/exp OR 'follow up'/exp OR 'case-control study':ab,ti OR 'studies, case-control':ab,ti OR 'study, case-control':ab,ti OR 'case-comparison studies':ab,ti OR 'case comparison studies':ab,ti OR 'case-comparison study':ab,ti OR 'studies, case-comparison':ab,ti OR 'study, case-comparison':ab,ti OR 'case control studies':ab,ti OR 'case control study':ab,ti OR 'studies, case control':ab,ti OR 'study, case control':ab,ti OR 'matched case-control studies':ab,ti OR 'case-control studies, matched':ab,ti OR 'case-control study, matched':ab,ti OR 'matched case control studies':ab,ti OR 'matched case-control study':ab,ti OR 'studies, matched case-control':ab,ti OR 'study, matched case-control':ab,ti OR 'cohort studies':ab,ti OR 'cohort study':ab,ti OR 'studies, cohort':ab,ti OR 'study, cohort':ab,ti OR 'retrospective studies':ab,ti OR 'studies, retrospective':ab,ti OR 'study, retrospective':ab,ti OR 'retrospective study':ab,ti OR 'longitudinal studies':ab,ti OR 'longitudinal study':ab,ti OR 'studies, longitudinal':ab,ti OR 'study, longitudinal':ab,ti OR 'longitudinal survey':ab,ti OR 'longitudinal surveys':ab,ti OR 'survey, longitudinal':ab,ti OR 'surveys, longitudinal':ab,ti OR 'follow-up studies':ab,ti OR 'follow up studies':ab,ti OR 'follow-up study':ab,ti OR 'studies, follow-up':ab,ti OR 'study, follow-up':ab,ti OR 'followup studies':ab,ti OR 'followup study':ab,ti OR 'studies, followup':ab,ti OR 'study, followup':ab,ti |
| #5 | #1 AND #2 AND #3 AND #4 |

**Web of Science：**

| Search Number | Search Description |
| --- | --- |
| #1 | TS=(Inflammatory Bowel Diseases OR Inflammatory Bowel Disease OR Bowel Diseases, Inflammatory OR Inflammatory Bowel Diseases OR Bowel Disease, Inflammatory OR Crohn Disease OR Ulcerative Colitis) |
| #2 | TS=(Child OR Children OR Pediatrics OR Pediatric OR Adolescent OR Adolescents OR Adolescence OR Adolescents, Female OR Adolescent, Female OR Female Adolescent OR Female Adolescents OR Adolescents, Male OR Adolescent, Male OR Male Adolescent OR Male Adolescents OR Youth OR Youths OR Teens OR Teen OR Teenagers OR Teenager) |
| #3 | TS=(Pulmonary Embolism OR Embolism, Pulmonary OR Embolisms, Pulmonary OR Pulmonary Embolisms OR Pulmonary Thromboembolisms OR Thromboembolisms, Pulmonary OR Pulmonary Thromboembolism OR Thromboembolism, Pulmonary OR Venous Thromboembolism OR Thromboembolism, Venous OR VTE OR Thromboembolism OR Thromboembolisms OR Venous Thrombosis OR Phlebothrombosis OR Phlebothromboses OR Thrombosis, Venous OR Thromboses, Venous OR Venous Thromboses OR Deep Vein Thrombosis OR Deep Vein Thromboses OR Thromboses, Deep Vein OR Vein Thromboses, Deep OR Vein Thrombosis, Deep OR Deep Venous Thrombosis OR Deep Venous Thromboses OR Thromboses, Deep Venous OR Thrombosis, Deep Venous OR Venous Thromboses, Deep OR Venous Thrombosis, Deep OR Deep-Vein Thrombosis OR Deep-Vein Thromboses OR Thromboses, Deep-Vein OR Thrombosis, Deep-Vein OR Deep-Venous Thrombosis OR Deep-Venous Thromboses OR Thromboses, Deep-Venous OR Thrombosis, Deep-Venous OR Thrombosis, Deep Vein OR DVT OR Thrombosis OR Thromboses OR Thrombus OR Blood Clot OR Blood Clots OR Atherothrombosis) |
| #4 | TS=(Case-Control Studies OR Cohort Studies OR Retrospective Studies OR longitudinal studies OR follow-up studies OR Case-Control Study OR Studies, Case-Control OR Study, Case-Control OR Case-Comparison Studies OR Case Comparison Studies OR Case-Comparison Study OR Studies, Case-Comparison OR Study, Case-Comparison OR Case Control Studies OR Case Control Study OR Studies, Case Control OR Study, Case Control OR Matched Case-Control Studies OR Case-Control Studies, Matched OR Case-Control Study, Matched OR Matched Case Control Studies OR Matched Case-Control Study OR Studies, Matched Case-Control OR Study, Matched Case-Control OR Cohort Study OR Studies, Cohort OR Study, Cohort OR Studies, Retrospective OR Study, Retrospective OR Retrospective Study OR Longitudinal Study OR Studies, Longitudinal OR Study, Longitudinal OR Longitudinal Survey OR Longitudinal Surveys OR Survey, Longitudinal OR Surveys, Longitudinal OR Follow Up Studies OR Follow-Up Study OR Studies, Follow-Up OR Study, Follow-Up OR Followup Studies OR Followup Study OR Studies, Followup OR Study, Followup) |
| #5 | #1 AND #2 AND #3 AND #4 |

**Cochrane Library:**

| Search Number | Search Description |
| --- | --- |
| #1 | MeSH descriptor: [Inflammatory Bowel Diseases] explode all trees |
| #2 | MeSH descriptor: [Crohn Disease] explode all trees |
| #3 | MeSH descriptor: [Colitis, Ulcerative] explode all trees |
| #4 | (Inflammatory Bowel Diseases):ab,ti,kw OR (Inflammatory Bowel Disease):ab,ti,kw OR (Bowel Diseases, Inflammatory):ab,ti,kw OR (Bowel Disease, Inflammatory):ab,ti,kw OR (Crohn Disease):ab,ti,kw OR (Ulcerative Colitis):ab,ti,kw |
| #5 | #1 OR #2 OR #3 OR #4 |
| #6 | MeSH descriptor: [Child] explode all trees |
| #7 | MeSH descriptor: [Pediatrics] explode all trees |
| #8 | (Child):ab,ti,kw OR (Children):ab,ti,kw OR (Pediatrics):ab,ti,kw OR (Pediatric):ab,ti,kw OR (Adolescent):ab,ti,kw OR (Adolescents):ab,ti,kw OR (Adolescence):ab,ti,kw OR (Adolescents, Female):ab,ti,kw OR (Adolescent, Female):ab,ti,kw OR (Female Adolescent):ab,ti,kw OR (Female Adolescents):ab,ti,kw OR (Adolescents, Male):ab,ti,kw OR (Adolescent, Male):ab,ti,kw OR (Male Adolescent):ab,ti,kw OR (Male Adolescents):ab,ti,kw OR (Youth):ab,ti,kw OR (Youths):ab,ti,kw OR (Teens):ab,ti,kw OR (Teen):ab,ti,kw OR (Teenagers):ab,ti,kw OR (Teenager):ab,ti,kw |
| #9 | #6 OR #7 OR #8 |
| #10 | MeSH descriptor: [Pulmonary Embolism] explode all trees |
| #11 | MeSH descriptor: [Venous Thromboembolism] explode all trees |
| #12 | MeSH descriptor: [Venous Thrombosis] explode all trees |
| #13 | MeSH descriptor: [Thrombosis] explode all trees |
| #14 | (Pulmonary Embolism):ab,ti,kw OR (Embolism, Pulmonary):ab,ti,kw OR (Embolisms, Pulmonary):ab,ti,kw OR (Pulmonary Embolisms):ab,ti,kw OR (Pulmonary Thromboembolisms):ab,ti,kw OR (Thromboembolisms, Pulmonary):ab,ti,kw OR (Pulmonary Thromboembolism):ab,ti,kw OR (Thromboembolism, Pulmonary):ab,ti,kw OR (Venous Thromboembolism):ab,ti,kw OR (Thromboembolism, Venous):ab,ti,kw OR (VTE):ab,ti,kw OR (Thromboembolism):ab,ti,kw OR (Thromboembolisms):ab,ti,kw OR (Venous Thrombosis):ab,ti,kw OR (Phlebothrombosis):ab,ti,kw OR (Phlebothromboses):ab,ti,kw OR (Thrombosis, Venous):ab,ti,kw OR (Thromboses, Venous):ab,ti,kw OR (Venous Thromboses):ab,ti,kw OR (Deep Vein Thrombosis):ab,ti,kw OR (Deep Vein Thromboses):ab,ti,kw OR (Thromboses, Deep Vein):ab,ti,kw OR (Vein Thromboses, Deep):ab,ti,kw OR (Vein Thrombosis, Deep):ab,ti,kw OR (Deep Venous Thrombosis):ab,ti,kw OR (Deep Venous Thromboses):ab,ti,kw OR (Thromboses, Deep Venous):ab,ti,kw OR (Thrombosis, Deep Venous):ab,ti,kw OR (Venous Thromboses, Deep):ab,ti,kw OR (Venous Thrombosis, Deep):ab,ti,kw OR (Deep-Vein Thrombosis):ab,ti,kw OR (Deep-Vein Thromboses):ab,ti,kw OR (Thromboses, Deep-Vein):ab,ti,kw OR (Thrombosis, Deep-Vein):ab,ti,kw OR (Deep-Venous Thrombosis):ab,ti,kw OR (Deep-Venous Thromboses):ab,ti,kw OR (Thromboses, Deep-Venous):ab,ti,kw OR (Thrombosis, Deep-Venous):ab,ti,kw OR (Thrombosis, Deep Vein):ab,ti,kw OR (DVT):ab,ti,kw OR (Thrombosis):ab,ti,kw OR (Thromboses):ab,ti,kw OR (Thrombus):ab,ti,kw OR (Blood Clot):ab,ti,kw OR (Blood Clots):ab,ti,kw OR (Atherothrombosis):ab,ti,kw |
| #15 | #10 OR #11 OR #12 OR #13 OR #14 |
| #16 | MeSH descriptor: [Case-Control Studies] explode all trees |
| #17 | MeSH descriptor: [Cohort Studies] explode all trees |
| #18 | MeSH descriptor: [Retrospective Studies] explode all trees |
| #19 | MeSH descriptor: [Longitudinal Studies] explode all trees |
| #20 | MeSH descriptor: [Follow-Up Studies] explode all trees |
| #21 | (Case-Control Studies):ab,ti,kw OR (Case-Control Study):ab,ti,kw OR (Studies, Case-Control):ab,ti,kw OR (Study, Case-Control):ab,ti,kw OR (Case-Comparison Studies):ab,ti,kw OR (Case Comparison Studies):ab,ti,kw OR (Case-Comparison Study):ab,ti,kw OR (Studies, Case-Comparison):ab,ti,kw OR (Study, Case-Comparison):ab,ti,kw OR (Case Control Studies):ab,ti,kw OR (Case Control Study):ab,ti,kw OR (Studies, Case Control):ab,ti,kw OR (Study, Case Control):ab,ti,kw OR (Matched Case-Control Studies):ab,ti,kw OR (Case-Control Studies, Matched):ab,ti,kw OR (Case-Control Study, Matched):ab,ti,kw OR (Matched Case Control Studies):ab,ti,kw OR (Matched Case-Control Study):ab,ti,kw OR (Studies, Matched Case-Control):ab,ti,kw OR (Study, Matched Case-Control):ab,ti,kw OR (Cohort Studies):ab,ti,kw OR (Cohort Study):ab,ti,kw OR (Studies, Cohort):ab,ti,kw OR (Study, Cohort):ab,ti,kw OR (Retrospective Studies):ab,ti,kw OR (Studies, Retrospective):ab,ti,kw OR (Study, Retrospective):ab,ti,kw OR (Retrospective Study):ab,ti,kw OR (Longitudinal Studies):ab,ti,kw OR (Longitudinal Study):ab,ti,kw OR (Studies, Longitudinal):ab,ti,kw OR (Study, Longitudinal):ab,ti,kw OR (Longitudinal Survey):ab,ti,kw OR (Longitudinal Surveys):ab,ti,kw OR (Survey, Longitudinal):ab,ti,kw OR (Surveys, Longitudinal):ab,ti,kw OR (Follow-Up Studies):ab,ti,kw OR (Follow Up Studies):ab,ti,kw OR (Follow-Up Study):ab,ti,kw OR (Studies, Follow-Up):ab,ti,kw OR (Study, Follow-Up):ab,ti,kw OR (Followup Studies):ab,ti,kw OR (Followup Study):ab,ti,kw OR (Studies, Followup):ab,ti,kw OR (Study, Followup):ab,ti,kw |
| #22 | #16 OR #17 OR #18 OR #19 OR #20 OR #21 |
| #23 | #5 AND #9 AND #15 AND #22 |
