# Supplementary material for: Increased risk of venous thromboembolism in children and teenagers with inflammatory bowel disease: a systematic review and meta-analysis
Source: PeerJ. 2026 Apr 1;14:e21056. doi: 10.7717/peerj.21056 (PMC13050219; doi:10.7717/peerj.21056)
Supplement: Supplemental Information 6 [file peerj-14-21056-s006.docx]

**Rationale:**

Patients with Inflammatory bowel disease (IBD) often have multiple extraintestinal manifestations, including venous thromboembolism (VTE).VTE has become the third leading cause of death from vascular diseases after heart attack and stroke, but it is not as well-known to the public as the former two. The patients with IBD often present with a hypercoagulable state, which will promote the formation of VTE. Research findings show that the relative risk (RR) of VTE in adults with IBD is twice that of the non-IBD population.At present, we know less about the VTE risk of IBD in children and teenagers. We conducted a systematic review and meta-analysis to consolidate recent evidence and clarify the relative risk of VTE in children and teenagers with IBD.

**Contribution:**

The previous meta-analyse has reported that the pooled incidence rate of VTE in children and teenagers with IBD is 0.02, the RR of VTE was found to be 2.99. However, in this study, only two studies were included when combining the RR, and no subgroup analysis was conducted. Our meta-analysis incorporated both previously omitted studies and newly published research, updating the RR of VTE in children and teenagers. Additionally, subgroup analyses were performed based on IBD type and VTE type. We found that the RR of VTE in children and teenagers with IBD may have been underestimated, and the results are influenced by the type of IBD and VTE. Compared with adults, we should also attach importance to the occurrence and prevention of VTE in children and teenagers.
